# The interaction between oral microbiota and gut microbiota in atherosclerosis

**DOI:** 10.3389/fcvm.2024.1406220

**Published:** 2024-06-12

**Authors:** Xinsi Li, Qian Li, Li Wang, Huifen Ding, Yizhong Wang, Yunfei Liu, Ting Gong

**Affiliations:** ^1^ Chongqing Key Laboratory of Oral Diseases and Biomedical Sciences, Chongqing Medical University, Chongqing, China; ^2^ Chongqing Municipal Key Laboratory of Oral Biomedical Engineering of Higher Education, Chongqing Medical University, Chongqing, China; ^3^ Department of Implantology, Stomatological Hospital of Chongqing Medical University, Chongqing, China; ^4^ Department of Prosthodontics, Stomatological Hospital of Chongqing Medical University, Chongqing, China; ^5^ Department of Research & Development, Zhejiang Charioteer Pharmaceutical Co., Ltd, Taizhou, China

**Keywords:** atherosclerosis, oral microbiota, gut microbiota, interaction, NO, LPS

## Abstract

Atherosclerosis (AS) is a complex disease caused by multiple pathological factors threatening human health-the pathogenesis is yet to be fully elucidated. In recent years, studies have exhibited that the onset of AS is closely involved with oral and gut microbiota, which may initiate or worsen atherosclerotic processes through several mechanisms. As for how the two microbiomes affect AS, existing mechanisms include invading plaque, producing active metabolites, releasing lipopolysaccharide (LPS), and inducing elevated levels of inflammatory mediators. Considering the possible profound connection between oral and gut microbiota, the effect of the interaction between the two microbiomes on the initiation and progression of AS has been investigated. Findings are oral microbiota can lead to gut dysbiosis, and exacerbate intestinal inflammation. Nevertheless, relevant research is not commendably refined and a concrete review is needed. Hence, in this review, we summarize the most recent mechanisms of the oral microbiota and gut microbiota on AS, illustrate an overview of the current clinical and epidemiological evidence to support the bidirectional connection between the two microbiomes and AS.

## 1 Introduction

Atherosclerosis, featured by cholesterol buildup and macrophage infiltration into arterial walls, is both metabolic and inflammatory in nature (1, 2). It is estimated that in 2020, 27.6% of samples aged 30–79 globally have an increased carotid intima-media thickness (IMT), which equals approximately 1,066.70 million individuals that are affected. This represents a 57.46% increase from 2000 (3). In recent years, studies on the pathogenesis of AS have emerged in an endless stream, among which studies on inflammasome have become a hot topic. Canakinumab Anti-inflammatory Thrombosis Outcome Study (CANTOS) and colchicine tests show that, Inflammatory bodies and their main product IL-1β play an important role in human atherosclerotic cardiovascular disease, which can exacerbate AS and plaque instability (4). On the NLRP3 inflammator-driven il-1 beta-IL-6 axis, residual risk from inflammation can be controlled by interfering with the co-stimulatory molecule CD40l-CD40 and selectively targeting tumor necrosis factor receptor associated factors (TRAFs), the control of inflammation triggers the occurrence of AS (5). In addition, Indoleamine 2, 3-Dioxygenase 1 (IDO1) deficition-mediated kynurenine deficiency exacerbates vascular smooth muscle cell calcification and plaque instability (6).

Since gut microbiota and oral microbiota have been confirmed to be related to inflammation and metabolism (1, 7, 8), plenty of studies have linked the two microbiomes to AS (9–11).

As early as a century ago, links between oral disease and cardiovascular disease (CVD) have been proposed (9, 12). Subsequent studies revealed an association between pathobionts from the oral cavity and AS (13, 14). For example, the burden of coronary AS was detected to be independently associated with self-reported oral diseases and tooth loss (15). In addition, patients with severe periodontitis are more likely to be accompanied with thicker carotid IMT (14). Besides, carotid atheromatous tissue obtained from oral-pathogens-infected individuals contained *P. gingivalis*-specific DNA (16). Although these studies support an association between periodontal pathogenic species and AS, specific elucidation of the mechanisms involved remains unknown.

Besides, a variety of previous researches have demonstrated that gut microbiota is also associated with AS. The gut microbiota can generate active metabolites, including trimethylamine N-oxide (TMAO) and short-chain fatty acids (SCFAs), which are beneficial to the regulation of hypertension and thrombosis. Moreover, by inducing systemic inflammation and influencing host lipid metabolism, gut microbiota may contribute to the progress of AS (8, 17, 18).

This review aims to investigate the connection between oral and gut microbiota on AS, including the influence of both microbiomes on the pathogenesis of AS, and how their interaction affects the occurrence of AS up to February of 2024.

## 2 Atherosclerosis

Contrary to the previous belief that AS is solely related to the accumulation of lipids in arterial vessels due to elevated circulating lipids, a new insight presents that the condition is characterized by a persistent inflammatory response (16). Infections enable the progression or development of AS happens by augmenting atherosclerotic alterations in vascular tissues (1). The multiple alterations encompass the increased absorption of cholesterol and modification of low-density lipoprotein (LDL) (19), the elevation of adhesion molecules and inflammatory protein expression (20, 21), and the promotion of blood clotting. Furthermore, specific pathogens involved in AS through accelerating the conversion of macrocytes into foam cells and triggering the release of cytokines that may result in plaque instability and even rupture, thus exerting atherosclerotic effects in macrophages (22, 23).

## 3 Oral microbiota and gut microbiota

### 3.1 Gut microbiota

Colonizing in the intestinal tract of humans, the gut microbiota is a vast and complex microbial community. Gut microbiota makes a crucial contribution to metabolism in the human body by producing enzymes that the human genome cannot encode, such as the synthesis of dietary carbohydrates, bile acids (BAs), and vitamins. Besides, in healthy individuals, gut microbiota may protect the permeability of the gastrointestinal mucosa, thereby supporting the function of the intestinal barrier to prevent LPS from entering the bloodstream from gut microbes (10). The gut microbiota present in the human body is mainly comprised of six phyla including *Firmicutes*, *Bacteroidetes*, *Proteobacteria*, *Actinobacteria*, *Verrucomicrobia*, and *Fusobacteria*, and the predominant types are *Bacteroidetes* and *Firmicutes* (18). A recent metagenome study divided gut microbiome into three clusters: *Bacteroides* >30% to enterotype I, *Prevotella* >15% to enterotype II, and the rest to enterotype “others” (III). This research discovered that the order Lactobacillales is remarkably enhanced while the phylum *Bacteroidetes* (*Bacteroides* + *Prevotella*) is declined among patients with coronary heart disease (17), suggesting a connection between gut microbiota and AS.

### 3.2 Oral microbiota

The microflora in the oral cavity mainly includes Firmicutes, Bacteroidetes, Proteobacteria, Fusobacteria, and Actinobacteria (10, 18). Oral microbiota has an impact on activating immune responses. For example, periodontitis-associated microbiota such as *Porphyromonas gingivalis* (*P. gingivalis*), a main pathogen causing periodontosis by initiating the immune response of the host (18). Furthermore, oral microbiota may participate in the progress of AS (24). A study examined the diversity of microbiota present in atherosclerotic plaque by utilizing 16S rRNA gene pyrosequencing. The results indicated that the combined abundances of Streptococcus and *Veillonella* in atherosclerotic plaque were closely linked to those in the oral cavity. Besides, the study found that some specific bacterial phylotypes were observed in both the atherosclerotic plaque and gut or oral samples obtained from the same individual (1). Numerous epidemiological researches have demonstrated that periodontal disease may elevate the risk of AS (25–27). Additionally, several studies have established a relationship between oral microbiota and the onset of AS (1, 10, 28).

## 4 The influence of oral microbiota on AS

Oral microbiota, beyond its role in the occurrence of periodontitis through dysbiosis, more crucial effects include contributing to CVDs, mechanisms are elaborated below (Table 1).

**Table 1:** Summary of the mechanisms by which oral microbiota affects AS.

Significant mechanism	Related target cell	Related signaling pathways
Invade cells	Vascular endothelial cells.	Enhance AS-related PAI-1 levels and biological activity (29). Alter the genes responsible for mitochondrial function (30, 31). Inhibit the down-regulation of genes in the PPAR-signaling pathway (32). Interference with reverse cholesterol transport (9).
Release lipopolysaccharide	Monocytes, macrophages, neutrophils, epithelial cells, and fibroblasts.	TLR4 (33). PAMPs (34). Form cytokines such as TNF-α, IL-1, IL-6, and IL-12 (35, 36).
Lipoprotein remodeling	Vascular endothelial cells, T-cell.	Ox-LDL issue in rapid absorption and cholesterol accumulation in the macrophagocytes (37). HDL stimulate eNOS generation and exhibit anti-inflammatory, antithrombotic, and anti-apoptotic properties (38).
Induce elevated levels of inflammatory mediators	Macrophage.	Levels of inflammatory mediators and acute phase proteins are increased (28). Adhesion molecules have raised (39, 40).
Intensify the immune response	Dendritic cells and macrophages.	MAMP-PRR (41).
Activate platelets	Not mentioned.	PAI-1 and other platelet activation markers are elevated (42, 43).
Stimulate enzymes’ production	Not mentioned.	MMP and MPO produce (44).

### 4.1 Invade cells

The impairment of endothelial function is an early sign of AS, and it can be initiated by various factors (45, 46), including the invasion of periodontal pathogens (24). At the DNA, RNA, or antigen level, various types of oral bacteria have been found in atheroma tissues (24). Live *P. gingivalis* and *A. actinomycetemcomitans* were detected in atheromatous tissue by Kozarov et al. (47). The genomic DNA of *A. actinomycetemcomitans*, *P. gingivalis*, and other microorganisms were first detected by using bacterial 16S rRNA-specific PCR analysis (48). Oral microbiota affects the occurrence and progression of AS by directly invading vascular endothelial cells and inducing endothelial dysfunction (ED) (24). Extensive research has been conducted on the mutual effect between *P. gingivalis* and cardiovascular cells (24). Adherence to and invasion of host cells by *P. gingivalis* is mediated by adhesins, including the major fimbriae (FimA) gingipains, and hemagglutinins (49, 50). In addition to adhesins, the adhesive and invasive capacity of *P. gingivalis* into endothelium is also facilitated by other microorganisms in the oral cavity, a case in point is Fusobacterium nucleatum (51). After *P. gingivalis* invades human aortic endothelial cells, it affects the occurrence and development of AS through several mechanisms. For example, inducing the pro-coagulant effects in endotheliocytes through enhancing AS-related plasminogen-activator inhibitor-1(PAI-1) levels and biological activity (29), eliciting a platelet aggregation response (52). Besides, altering the genes responsible for mitochondrial function in endothelial cells may lead to mitochondrial dysfunction (30, 31). Inhibiting the down-regulation of genes in the PPAR-signaling pathway may promote the development of AS by preventing plaque regression (32). Additionally, interference with reverse cholesterol transport by *P. gingivalis* may also impede the regression of atherosclerotic plaque (9).

### 4.2 Release LPS

As a crucial virulence factor of gram-negative microbiota, LPS, commonly known as endotoxin, are responsible for systemic endotoxemia when it translocating to the bloodstream (53). The bioactive lipid A of LPS, either as individual molecules or in aggregates, is identified by the LPS receptor complex, which is composed of toll-like receptor 4 (TLR4), cluster of differentiation 14 (CD14), and myeloid differentiation 2 (MD-2). This complex is expressed in a variety of cells, such as monocytes, macrophages, neutrophils, epithelial cells, and fibroblasts (10). Lipid A of *P. gingivalis* mainly binds to TLR4 and is either inert or antagonistic in response to TLR4 activation (33). This activation mediates systemic inflammatory response, affects the vessel walls and atheromatous lesions (54). Endotheliocytes in the vasculature are prone to AS and can be activated by LPS originating from periodontal pathogens, resulting in the adhesion of monocytes. What's more, LPS is one of the main types of pathogen-associated molecular patterns (PAMPs) that are known to elicit severe immune reactions in the event of a pathogen trespassing the epithelial barrier and reaching the bloodstream (34). The relevant immune response is related to the development of AS. Additionally, LPS can stimulate host cells to form cytokines such as TNF-α, IL-1, IL-6, and IL-12, which in turn increase leukocyte adhesion to the endothelium and vascular permeability (35, 36). As a result of vascular inflammation, coagulation may be activated, which can potentially lead to thrombosis (55). What's more, LPS may promote foam cell formation and LDL accumulation (56). Besides, LPS also stimulates the MMP's production in endothelial cells, macrophages, and mast cells within plaques, giving rise to the rupture of fibrous caps in plaques (57–59).

### 4.3 Lipoprotein remodeling

Owing to the abundance of gram-negative bacteria in the subgingival microbiota, individuals suffering from periodontitis experience endotoxemia (60). Endotoxemia can contribute to the formation of AS by elevating LDL concentrations and reducing high-density lipoprotein (HDL) concentrations. In a meta-analysis, individuals with periodontitis have been implicated in having significantly elevated LDL and reduced HDL in comparison with the normal group (7). Endotoxemia correlates with a predominance of the LDL phenotype, which is highly proatherogenic as it facilitates their accumulation within the macrophages and promotes the form of subendothelial foam cells—a signal of early AS (54, 61, 62). Small, dense LDL fragments decrease their affinity to the LDL receptor, allowing them to easily penetrate the arterial wall, and undergo oxidative modifications (ox-LDL), issuing in rapid absorption and cholesterol accumulation in the macrophagocytes (37). Furthermore, oligopeptide fragments of the LDL protein, apoB100, can function as autoantigens, triggering T-cell activation and adaptive immune responses that promote inflammation and macrophage activation (54). HDL is regarded as anti-atherosclerotic owing to characteristics beyond its role in reverse cholesterol transport, other protective effects include stimulating endothelial nitric oxide synthase (eNOS) generation and exhibiting anti-inflammatory, antithrombotic, and anti-apoptotic properties, which may directly safeguard the endothelial cells (38).

### 4.4 Induce elevated levels of inflammatory mediators

Endothelial cell dysfunction may be indirectly induced in patients with periodontal infection through a systemic inflammatory state. Levels of inflammatory mediators and acute phase proteins including TNF-α, IL-1, IL-6, IL-8, IFN, and C-reactive protein (CRP) are increased following blood exposure to bacterial antigens (28). CRP can directly impact vascular vulnerability via multiple mechanisms, such as modulating the localized expression of adhesion molecules, reducing endothelial nitric oxide (NO) bioactivity, and modifying LDL uptake by macrophages (18). Ishikawa et al. proposed that CRP within atherosclerotic plaques directly contributes to the inflammatory process of AS (63). Once in circulation, these mediators can participate in the immune alteration of endotheliocytes from an antithrombotic to a prothrombotic state, which contributes to formulating atherosclerotic lesions (64, 65). What's more, researchers have revealed that respondents with periodontitis have raised adhesion molecules including macrophage chemoattractant peptide-1(MCP-1), macrophage colony stimulating factor (MCSF), E-selectin, ICAM-1, and VCAM-1 in comparison with the normal group (39, 40). These inflammatory cytokines have been linked to ED and the progression of atheromatous plaques in several observational studies (44). Among them, MCP-1 is responsible for recruiting monocytes, MCSF converts monocytes into macrophages, and macrophages eventually transform into foam cells, thereby inducing ED and triggering AS (66). E-selectin acts as a receptor for carbohydrate ligands on leukocyte surfaces. Its role is to attract circulating leukocytes and bind them to locate at the surface of endothelial cells. Following this, the transmembrane glycoprotein ICAM-1 engages with integrins on leukocyte surfaces, facilitating robust adhesion to the endothelium. Consequently, this process contributes to AS via the inflammatory effects on the vascular endothelium (44).

### 4.5 Intensify the immune response

*P. gingivalis* can exist in immune cells, including dendritic cells and macrophages, escape the immune reaction, and enable the bacteria to spread, thus bacteremia and endotoxemia may occur (67). The detection of bacteremia and endotoxemia is primarily carried out by host cells through the signaling of microorganism-related molecular patterns-pattern recognition receptors (MAMP-PRR). These include the peptidoglycan receptor nucleotide-binding oligomerization domain-containing protein 1 (NOD1), diacylated and triacylated lipopeptides receptors TLR2 and TLR6, and CpG oligodeoxynucleotides receptor TLR9. Direct engagement of PRR by MAMP contributes to the development of AS (41).

### 4.6 Activate platelets

A report has revealed that patients with infectious agents possess advanced plasma fibrinogen, which can stimulate the increase of protein that is inflamed and elicit blood clotting (68). Furthermore, elevated PAI-1 was found in samples with severe periodontitis (69). PAI-1, a protease inhibitor, reduces fibrinolysis by inhibiting tPA and uPA, indirectly promoting blood clotting (70). Other platelet activation markers, such as CD18, sP-selectin, P-selectin, and activated glycoprotein IIb/IIIa, were found to have a connection with periodontitis and AS. Research has demonstrated that platelets contribute to atheroma formation and thrombosis due to their aggregation and pro inflammatory mediator release after activation (42, 43).

### 4.7 Stimulate enzymes' production

Oral bacteria induce the production of matrix metalloproteinases (MMP) and myeloperoxidase (MPO) (28, 44). MMP can trigger atherosclerotic plaque rupture by degrading collagen in fibrous caps (71). The enzyme MPO, whose substrate can be inflamed and oxidized, has been linked to atheroma plaque development and suppresses the expression of endothelial effect in numerous observational studies (44).

## 5 The influence of gut microbiota on AS

The gut microbiota has a significant impact on sustaining our health in the human body (18). However, when it comes to dysbiosis, it might lead to an inflammatory response, which could promote the occurrence of AS (72). The main mechanisms are shown below (Table 2).

**Table 2:** Summary of the mechanisms by which gut microbiota affects AS.

Significant mechanism	Related target cell	Related signaling pathways
Produce TMAO	Macrophages, Platelet, Vascular endothelium Hepatocytes	Disturbance of bile acid metabolism Inhibition of the RCT Inducement of foam cell formation, Activation of platelets Endothelial inflammatory injury (73)
Produce SCFAs	T cell Treg cell	Regulation of blood pressure by binding SCFA binding G protein-coupled receptor, Reducing vascular inflammation and atherosclerotic lesion burden (74, 75)
Regulate BA	Hepatocytes Macrophages T cell Vascular endothelial cell Vascular smooth muscle cells	bacterial enzymes for the generation of secondary BAs FXR-SHP pathway by LXR and SREBP-1c FXR and TGR5 Regulation of immune function: Impaired gut integrity promoting systemic inflammation Activation of BARs (FXR, TGR5, and VDR) and MRs (76)
Release LPS	Macrophages Hepatocytes Intestinal neuroendocrine L-cells	Recognized by TLR4 Down-regulate expression of LXR-á up-regulate expression of proatherogenic LDL receptor, VLDL receptor, and AdipoR2. (77, 78)
Stimulate immune response	Macrophages Intestinal epithelial cell Immune cell	TLR signaling Chylomicron-facilitated transport Extracellular leakage through tight junctions in the epithelial lining (77, 79)

RCT, reverse cholesterol transport; LXR, X receptor; SHP, short heterodimer partner; SREBP-1c, sterol regulatory element binding protein 1c; FXR, farnesoid X receptor; TGR5, Takeda G protein-coupled receptor; MR, muscarinic receptor; VDR, vitamin D receptor; BARs, BA receptors; TLR, Toll-like receptor; LXR, transcription factor liver X receptor; LDL, low density lipoprotein; VLDL, very low density lipoprotein; AdipoR2, adiponectin receptor 2.

### 5.1 Produce active metabolites

#### 5.1.1 TMAO

Gut microbiota has a great influence on human metabolism (72). With the assistance of gut microbiota, the metabolism of choline, phosphatidylcholine, and carnitine can be transformed to produce trimethylamine (TMA), which is subsequently modified into TMAO by heparin-containing monooxygenase (FMO). Emerging evidence from clinical trials suggests that TMAO can not only impact cholesterol levels but also has a potential association with early AS (73). TMAO impacts various metabolic processes in the human body, including the metabolism of cholesterol, oxidant stress, immune regulation, and inflammatory response. In the circulatory system, TMAO can inhibit the synthesis of BA and reverse the transportation of cholesterol, leading to the buildup of lipids in macrophages. Eventually, these macrophages turn into foam cells, which contribute to the development of plaques in vessels (17). Moreover, in endothelial cells, TAMO can activate MAPK and NF-*κ*B signaling pathways, causing vascular inflammation and increasing the formation of thrombus (18, 80).

#### 5.1.2 SCFAs

In the human gastrointestinal tract, some indigestible polysaccharides and proteins can be metabolized into SCFAs by gut microbiota, including acetate, propionate, butyrate acid, and formate (9). Epidemiological studies have indicated that a higher dietary fiber intake is closely related to a reduced incidence of hypertension, and SCFAs are beneficial to prevent cardiovascular damage (74). Among them, propionate can inhibit Acetyl-coenzyme A synthetases (ACS) and lower cholesterol levels; butyrate can promote the differentiation of Treg, increase Foxp3 promoter, affect cell G1 phase cyclin, control the propagation of vascular smooth muscle cells, and thus restrain myocardial fibrosis (81). Moreover, butyrate is also beneficial for intestinal epithelial cells (IECs) to maintain intestinal barrier integrity (72).

### 5.2 Regulate BA

Gut microbiota is capable of adjusting the BAs synthesis, the final metabolite of cholesterol, which may induce AS (1). Cholesterol is synthesized into cholic acid and chenodeoxycholic acid in the liver. Subsequently, in the distal ileum, the process of converting cholic acid into deoxycholic acid and chenodeoxycholic acid into lithocholic acid is facilitated by the gut microbiota. BAs are generated by the liver and reserved in the gallbladder. During eating, they are delivered to facilitate the intestinal absorption of various compounds, including triglycerides, cholesterol, and fat-soluble vitamins. About 95% of BAs are reabsorbed by the intestinal wall and transported back to the liver, forming a process known as hepato-enteric circulation. BAs can play a role in lipid homeostasis, immunity, and heart function. During this process, gut microbiota can activate signaling pathways that regulate the metabolic levels of total BA, deoxycholic acid, and lithocholic acid, which may influence the development of AS (76).

### 5.3 Release LPS

Studies have shown that LPS can trigger inflammatory responses by activating the TLRs pathway, inducing vascular oxidative stress, ED, and vascular inflammation (77, 82). These effects of LPS can accelerate the formation of plaques and thrombosis. Besides, the lipid A structure of LPS vary from diverse types of microbiomes, resulting in different biological activities (18, 78).

### 5.4 Stimulate immune response

Gut microbiota can engage with the immune system by inducing immune tolerance and reducing inflammation (77, 79). The innate immune system is responsible for detecting and eliminating pathogenic microbes to reduce inflammation, which is accomplished through the secretion of various factors such as secretory immunoglobulin A (SIgA), TLR5, and inflammasome (18, 83). In adaptive immunity, gut microbiota can participate in mediating neutrophil migration and affecting the T cells' maturation cycle. However, the activation of the immune system induced by pathogenic microorganisms developed in a diet high in salt or fat with inadequate fiber may increase the risk of AS. This effect occurs by stimulating the generation of Th17 cells, which then migrate to damage vascular function and water-salt balance (84).

## 6 Interaction between oral bacteria and gut bacteria

### 6.1 Oral microbiota causes an imbalance of gut microbiota, and periodontal inflammation exacerbates gut inflammation

Oral microbiota may cause variations to the gut microbiota, contributing to a concurrent increase in proinflammatory cytokines and gut permeability that are followed by systemic disease progression (10, 85, 86). The component of the gut microbiota was changed after oral gavage of *P. gingivalis in vivo*. Specifically, in the ileal contents, there was an elevation in the percentage of *Bacteroides* and a decrease in *Firmicutes* (87). This observation suggests that the invasion of oral microbiota can potentially impact gut microbiota by promoting the proliferation of oral pathobionts including *Klebsiella/Enterobacter* species (88). Amassed oral pathobionts migrate to the lower gastrointestinal tract and are ectopically located in the intestine through the enteral route or hematogenous route (88, 89), where they activate the inflammasome-mediated IL-1 signaling in colonic mononuclear phagocytes, causing inflammation (88). Oral pathobiont *K. aerogenes* likely induces IL-1b production via caspase-11-mediated, non-canonical inflammasome activation, therefore exacerbate colitis (88, 90).

The presence of periodontitis can increase the production of Th17 cells in the mouth. These cells have an affinity for the gut and migrate there in the presence of inflammation, which establishes a connection between oral and gut health (88). Once in the intestine, these Th17 cells are stimulated by periodontal pathogens that have translocated, leading to the occurrence of colitis. What is noteworthy is that these cells are not activated by commensal gut microbes (88, 91). Periodontitis links oral and intestinal health through the introduction of pathogenic T cells and colitogenic pathobionts that aggravate intestinal inflammation, creating a dual mechanism involving both microbial and immune systems (88).

However, oral pathobionts do not colonize the gastrointestinal tract of healthy individuals. In order for oral pathobionts to successfully colonize the gut, two conditions need to be met: disruption of colonization resistance of gut-resident microbiota and oral inflammation. The first condition involves the disruption of colonization resistance. This resistance is normally provided by the resident microbiota in the gut, which prevents the invasion of foreign microbes. In the presence of gut inflammation, this resistance is perturbed, allowing ingested oral pathobionts to outcompete and displace the resident bacteria. The inflammatory milieu in the gut also provides a favorable environment for the growth of Enterobacteriaceae, including bacteria translocated from the oral mucosa. However, it is important to note that the loss of colonization resistance or gut inflammation alone is not sufficient to facilitate ectopic gut colonization by oral pathobionts (90, 92, 93). The second condition that must be met is oral inflammation. There appears to be a threshold for the number of ingested oral pathobionts that must be reached for them to successfully transition from the oral cavity to the gut. Periodontal inflammation plays a crucial role in raising the abundance of oral pathobionts and increasing the likelihood of their successful passage through the acidic environment encountered in the stomach. In summary, both gut inflammation and oral inflammation are necessary for oral pathobionts to colonize the gut. Gut inflammation disrupts colonization resistance, allowing the oral microbes to invade the gut, while oral inflammation increases the abundance of oral pathobionts and facilitates their transition from the oral cavity to the gut (94, 95).

### 6.2 Gut and oral microbiota work together to regulate AS

#### 6.2.1 Modulate the NO pathway

**Figure 1:**
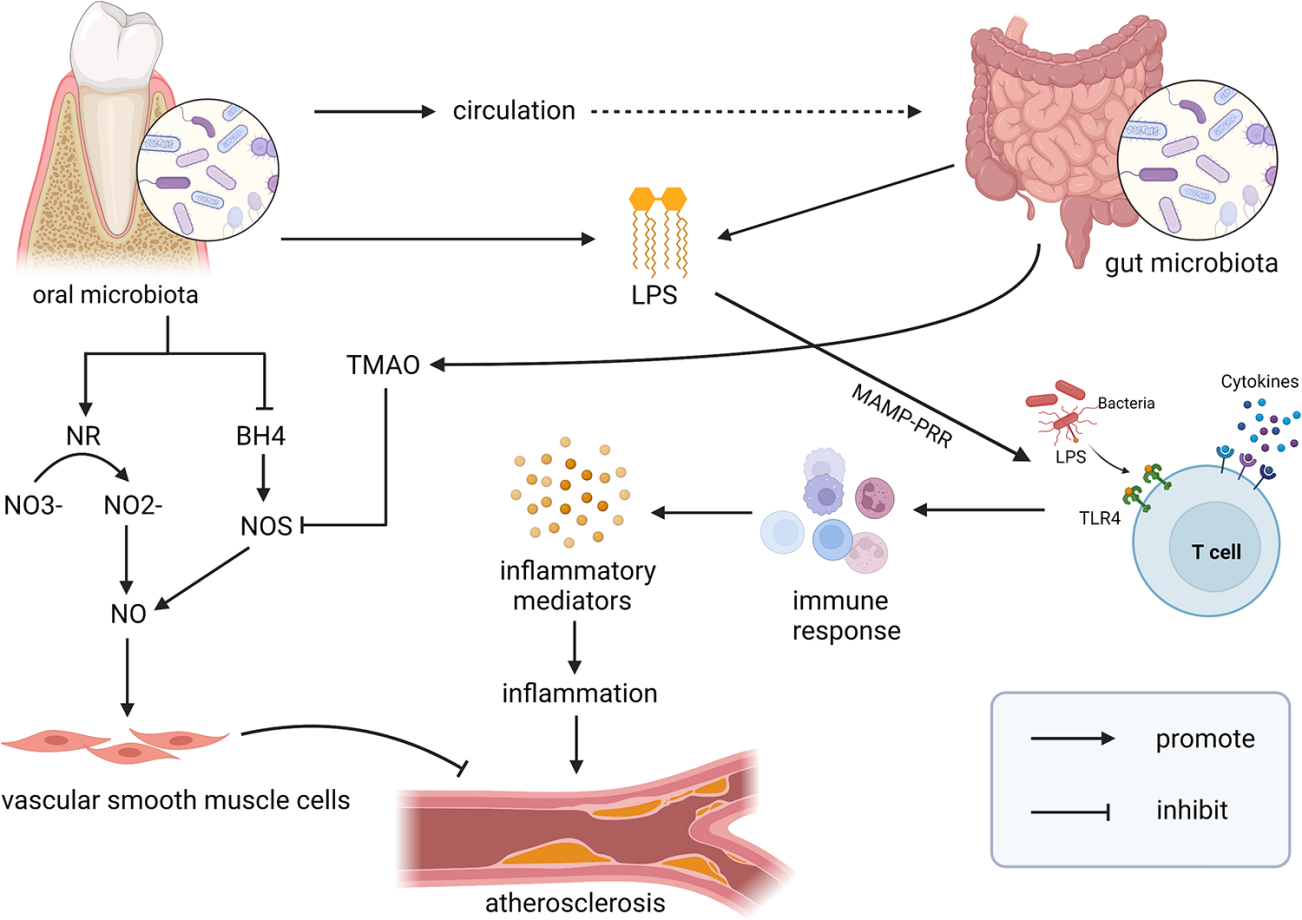
Multiple pathways linking the interaction between oral and gut microbiota on AS. Oral microbiota and gut microbiota are shown to be associated with the progress of AS. They act synergistically to regulate AS by modulating NO pathway, releasing LPS, stimulating immune response. Moreover, there is evidence that oral microbiota may circulate to the gut, where they interact and further exacerbate the development of AS. TMAO, trimethylamine N-oxide; BH4, tetrahydrobiopterin; NR, nitrite reductase; NO, nitric oxide; NOS, nitric oxide synthetase; LPS, lipopolysaccharide; MAMP-PRR, molecular patterns-Pattern recognition receptors; TLR4, toll-like receptor 4. Created with BioRender.com.

As a critical signaling molecule, NO performs a crucial function in the human cardiovascular system. Synthesized by endothelial cells, NO can induce vascular smooth muscle relaxation, thus dilating blood vessels. Besides, it inhibits smooth muscle cell proliferation, platelet aggregation, and oxidative stress, so NO deficiency may lead to AS (96, 97). Oral microbiota can assist in the production of the NO to a certain extent. Salivary glands absorb 25% of nitrate in food, and nitrate is formed by endogenous oxidation. Symbiotic bacteria in the mouth produce nitrate reductase, metabolize NO3− in saliva to NO2−, and help the host complete the first step of the NO conversion process (98). Another study has shown that periodontal pathogens can reduce the synthesis of NO and tetrahydrobiopterin (BH4) in mice, which is a cofactor of nitric oxide synthetase (NOS), and its reduction will lead to a decrease in NOS and NO synthesis in blood vessels and colon (99). In addition, studies have indicated that TMAO can facilitate the expression of superoxide ions and pro-inflammatory cytokines, reduce the activity of eNOS and decrease the bioavailability of NO in aortic and vascular endothelial cells (72). Therefore, oral and gut microbiota have a combined effect on AS by regulating NO production (Figure 1).

#### 6.2.2 Release LPS

As a major constituent of the envelope of gram-negative bacterial cells, LPS released by oral and gut microbiota could produce diverse activity to induce systemic and vascular inflammation, which is a key factor that promotes the maturation and rupture of plaque (Figure 1) (100). LPS can react with numerous types of host cells, such as neutrophils, macrophages, monocytes, fibroblasts, and epithelial cells to trigger the expression of inflammatory mediators and activate endothelial cells, ultimately causing “metabolic endotoxemia” defined by low-grade inflammation, insulin resistance, and an increase of AS (10, 82). Binding with TLR4, LPS can trigger the differentiation and activation of macrophages and the secretion of inflammatory cytokines for mediating the inflammatory response (54, 82). Besides, LPS may promote foam cell formation and LDL accumulation, and ox-LDL can upregulate TLR4 expression in macrophages, further promoting the development of inflammation and AS (101, 102).

#### 6.2.3 Stimulate immune response

Both oral and gut microbiota can play a part in the immune system to stimulate immune response (Figure 1) (18, 24). Some oral pathogens can survive in host immune cells, evade the immune response, spread in blood, cause bacteremia and endotoxemia, which may facilitate the generation of inflammatory cytokines and promote AS through MAMP-PRR pathways (41, 103). The healthy gut microbiota can induce immune tolerance and relieve inflammation by mediating neutrophil migration and affecting the differentiation and maturation of T cells, while under certain risk conditions, pathogenic microorganisms in the gut may activate the immune system and promote AS. They can motivate the production of Th17 cells, and accelerate the expression of cytokines and chemokines to induce an inflammatory response, which may alter blood vessel function and contribute to AS (18, 104) (Table 3).

**Table 3:** Treatment methods associated with microbiota on AS.

Method	Mechanism	Reference
Dietary intervention	The habitual diet that are coherent with the features of the traditional Mediterranean diet preventing AS	(105–107)
Probiotics and prebiotics	Suppressing oxidative stress, improving immunomodulation, and correcting lipid, glucose, and cholesterol metabolism	(108, 109)
Enzyme inhibitors	Reducing the generation of TMA and TMAO in the body	(73, 110)
Regulate intestinal immunity	Oral immune modulation, inducing Th3 and Foxp3 + Treg, inhibiting the formation of AS through a TGF-β-dependent mechanism, etc.	(72, 111)

TMA, trimethylamine; TMAO, trimethylamine N-oxide; TGF, transforming growth factor.

## 7 Treatment methods

### 7.1 Dietary intervention

Dietary interventions are an effective way to control the microbiota in our bodies. The food we eat is also the food of bacteria, so we can choose the types of bacteria by changing our diet (105). An experimental study demonstrated that dietary fats and oral infection may significantly contribute to the dysbiosis of gut microbiota related to AS by reduced community complexity and excessive growth of specific symbiotic microbiota. And after oral infection with *P. gingivalis*, the diet-induced lipid-lowering effect to control plaque accumulation was attenuated (112). Furthermore, high-salt diets could enhance local and systemic inflammation by elevating pro-inflammatory cytokines and altering intestinal permeability, some of which may come from specific microbiota (75). Some studies have shown that Mediterranean diet affects the microbiota and the relevant metabolism (106). This dietary pattern was related to a lower proportion of *Bacteroidetes*, *Firmicutes*, *Streptococcus* and higher *Bifidobacterium*, *Catenibacterium* and fecal SCFAs (75). The high-fiber diet is beneficial for cardiovascular health (107).

### 7.2 Probiotics and prebiotics

Probiotics have great potential for treating various diseases by promoting nutrient absorption and regulating the balance of gut microbiota. Studies have found that the *Bifidobacterium* can inhibit the development of AS to a certain extent, so it is possible to treat AS by using probiotics to maintain the balance of gut microbiota (108). Prebiotics are a kind of organic matter that can provide nutrients to probiotics, promote their reproduction, and regulate the composition and function of microbiota. At the same time, prebiotics improves glucose tolerance and blood lipid (109).

### 7.3 Enzyme inhibitors

Many experiments have demonstrated that the TMAO pathway is associated with AS, so microbial enzymes that produce TMA during metabolism may be a potential target (73). By developing enzyme inhibitors that restrain the activity of related enzymes, the levels of TMA and TMAO in the body can be reduced, thus reducing the risk of AS (110).

### 7.4 Regulate intestinal immunity

Studies have indicated that oral administration of anti-CD3 antibody therapy can induce Th3 and Foxp3 + Treg, inhibiting the formation of AS through a TGF-β-dependent mechanism (111). Using the gut microbiota as a therapeutic target to prevent AS, regulating the Treg and DCs in intestinal immunity may serve as a new strategy to prevent AS (8, 72). Another study reported a significant negative correlation between the abundance of the *Bacteroides* genus and fecal LPS levels. And there might be a novel therapeutic strategy for preventing AS through increasing *Bacteroides* abundance to reduce fecal LPS levels and suppress immune responses (78).

## 8 Conclusion

Both oral microbiota and gut microbiota can influence the development of AS through various mechanisms. Commonly shared pathways between oral and gut microbiota may include modulating NO pathway, releasing LPS, and stimulating the immune response. Through these shared pathways, gut and oral microbiota may interact with each other and promote the occurrence of AS. For example, pathogenic bacteria in the oral cavity can enter the systemic circulation and collaborate with bacteria in the gut to promote fatty acid oxidation, abnormal lipid metabolism, and inflammation. Additionally, the oral and gut microbiota can jointly induce autoimmune and inflammatory responses, accelerating the progression of AS. Therefore, maintaining a healthy oral and gut microbiota is crucial in preventing the occurrence of AS.

Healthy microbiota can maintain the body's homeostasis and inhibit disease development. However, microbiota dysbiosis may lead to the disorder of metabolism, induce inflammation, and promote vascular injury and the development of AS. In addition, there may be an interactive pathway between oral and gut microbiota that can promote AS further, but the specific mechanism remains unclear. Therefore, intensive research about the metabolic pathway between oral and gut microbiota and their interaction mechanism on AS may provide a method for predicting and treating AS.

In the future, a large number of cohort studies are needed to understand the function of specific microbial pathways and their metabolites and to develop predictive models of AS. Further research on next generation sequencing and metagenomics will be conducted to provide data that can guide clinical work. We hope to find identify specific pathways by which oral microbiota influences gut microbiota and metabolism to better prevent AS through innovative strategies based on microbiota interactions.
